# Prognostic value of platelet recovery degree before and after achieving minimal residual disease negative complete remission in acute myeloid leukemia patients

**DOI:** 10.1186/s12885-020-07222-4

**Published:** 2020-08-05

**Authors:** Yang Wang, Hua Wang, Weida Wang, Wenjian Liu, Nawei Liu, Shuang Liu, Yue Lu

**Affiliations:** 1 Department of Hematologic Oncology, Sun Yat-sen University Cancer Center, 651 Dongfengdong Rd, Guangzhou, 510060 China; 2 State Key Laboratory of Oncology in Southern China, Collaborative Innovation Center for Cancer Medicine, Guangzhou, 510060 China

**Keywords:** Acute myeloid leukemia (AML), Complete remission (CR), Platelet recovery degree, Progression free survival (PFS), Overall survival (OS)

## Abstract

**Background:**

Risk stratification and prognosis prediction of acute myeloid leukemia (AML) are largely dependent on pre-treatment information. However, post-treatment data also provides much useful information. In this retrospective study, we explored whether the level of blood count recovery before and after the first minimal residual disease (MRD) negative complete remission (CR) is relevant to clinical outcomes of AML patients.

**Methods:**

For each included patient, peripheral platelet counts were measured on the day before initial treatment (PLT_pre_), whereas platelet peak values (PLT_peak_) were recorded after marrow recovery following the chemotherapy course inducing the first MRD-negative CR. The difference (D_PLT_) between these two values (D_PLT_ = PLT_peak−_PLT_pre_) was calculated. X-tile software was utilized to establish the optimal cut-point for D_PLT_, which was expected to distinguish CR patients with different clinical outcomes. A cross validation analysis was conducted to confirm the robustness of the established cut-point. The results were further tested by a Cox multivariate analysis.

**Results:**

The optimal cut-point of D_PLT_ was determined as 212 × 10^9^/L. Patients in high D_PLT_ group were observed to have a significantly better PFS (*p* = 0.016) and a better OS (without statistical significance, *p* = 0.106). Cox multivariate analysis showed that higher D_PLT_ was associated with longer PFS (HR = 2.894, 95% CI: 1.320–6.345, *p* = 0.008) and longer OS (HR = 3.077, 95% CI: 1.130–8.376, *p* = 0.028).

**Conclusion:**

Platelet recovery degree before and after achieving MRD-negative CR (D_PLT_) is a potential predictor of clinical outcomes in CR patients. Higher D_PLT_ value is associated with longer PFS and OS. Our findings may help to develop simple methods for AML prognosis evaluation.

## Background

Acute myeloid leukemia (AML) is a highly heterogeneous disease, for which precise diagnosis and risk stratification is crucial. Currently the best diagnostic methods for AML include morphologic, flow cytometric, cytogenetic and molecular examination (sometimes bone marrow biopsy is also needed). A combination of these examinations presents a relatively comprehensive understanding of the disease [1, 2]. On the other hand, discovering new biomarkers associated with prognosis and exploring their relationship with clinical outcomes have become the focus of researches. In addition to pre-treatment information, some types of post-treatment information, such as minimal residual disease (MRD) [3, 4], response to first induction chemotherapy [5], and repeated bone marrow examination (morphologic, cytogenetic, flow cytometric, or molecular) during or after treatment courses [6–8] are also reported to be informative. As the embodiment of “real response” to standard therapy, post-treatment data should be carefully considered. It is a necessary complement for prognosis evaluation. For example, although more than 50% of newly diagnosed AML patients will achieve CR after standard induction chemotherapy, a large proportion of them will relapse and eventually die after different durations of CR [9]. Thus, the predictive factor of this difference among CR patients should be further identified. In this study, patients achieving MRD-negative CR were included. We calculated the platelet count changes between ‘before treatment’ and ‘after achieving the first MRD-negative CR’ (D_PLT_) for each patient, and explored whether or not this platelet change is potentially predictive of progression-free survival (PFS) or overall survival (OS) in these patients.

## Methods

### Patients and methods

A total of 105 patients newly diagnosed with AML (M3 and myeloid sarcoma excluded) in the Sun Yat-sen University Cancer Center from 2002.10 to 2016.12 were enrolled in this study. The diagnosis was based on the 2002 World Health Organization (WHO) criteria [10]. All patients received standard induction chemotherapy containing cytarabine and DNR/IDR (DNR 50 mg/m^2^, d1–3 + Ara-C 100 mg/m^2^, d1–7; or IDA 12 mg/m^2^, d1–3 + Ara-C 100 mg/m^2^, d1–7). Among the 105 patients, 38 never achieved MRD-negative CR or died of complications in induction chemotherapy courses, while the other 67 obtained MRD-negative CR after one, two or three courses of induction therapy. MRD was detected with multiparametric flow cytometry (MFC) [7]. The sensitivity of the MRD assay was 0.01%. All patients who achieved MRD-negative CR also met the criteria of the widely used morphological definition of CR: the presence of < 5% blasts in the bone marrow, the absence of extramedullary disorders, the recovery of neutrophil counts > 1 × 10^9^/L, and the platelet count > 100 × 10^9^/L in the peripheral blood. All of these 67 patients then acquired consolidation therapy, among whom 32 received JALSG AML-201 regimen [11], 24 received AML-87 regimen [12], and 11 received High Dose Ara-C regimen [13]. After consolidation therapy was accomplished, follow-up was conducted and prognostic information (PFS and OS) was obtained. This study was approved by the Ethical committee of Sun Yat-sen University Cancer Center. Informed consent was obtained from all individual patients included in the study. All experiments were performed in accordance with the ethical standards of Declaration of Helsinki.

**Fig. 1:**
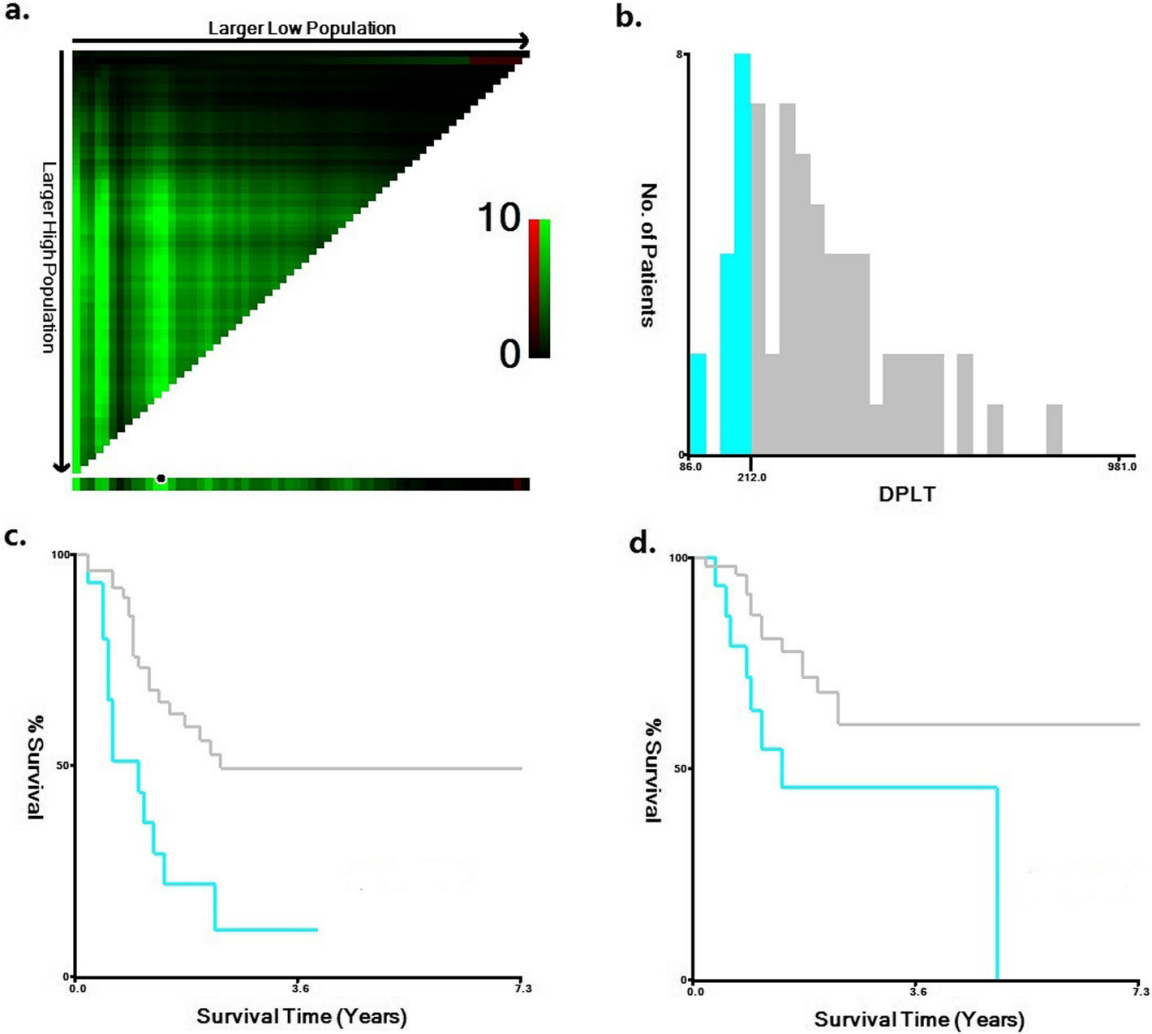
X-tile analysis of survival data of 67 included patients. Every pixel of the plot represents the χ^2^ log-rank value generated from corresponding division of the data. Direct associations between D_PLT_ and survival (higher D_PLT_ indicates a better outcome) are colored green, whereas indirect associations (higher D_PLT_ indicates poorer outcome) are colored red. A brighter (green or red) pixel color represents a larger χ^2^ log-rank value and a stronger association. **a.** Determination of the optimal cut-point. Survival data used for cut-point optimization is PFS. The optimal cut-point (D_PLT_ = 212 × 10^9^/L) appears at the brightest pixel (The black dot on the straight line below). **b.** A histogram showing the distribution of the entire cohort and the established cut-point. **c.** Kaplan-Meier curve of PFS between high and low D_PLT_ groups. The cutoff value is D_PLT_ = 212 × 10^9^/L. A significantly better PFS is observed in high D_PLT_ group (*p*-value given by cross-validation is 0.016. The blue line represents for low D_PLT_ group while the gray line represents for high D_PLT_ group) **d**. Kaplan-Meier curve of OS between high and low D_PLT_ groups. The cutoff value is D_PLT_ = 212 × 10^9^/L. A better OS is observed in high D_PLT_ group, but without significance (*p* value given by cross validation is 0.106. The blue line represents for low D_PLT_ group and the gray line represents for high D_PLT_ group)

For each of the 67 MRD-negative CR patient, we measured peripheral platelet count on the day before initial treatment (denoted as PLT_pre_), and platelet peak value after marrow recovery following the chemotherapy course that induced the first MRD-negative CR (denoted as PLT_peak_). PLT_peak_ appeared during 28–35 days from the first day of the chemotherapy course in all enrolled patients. Then the difference (denoted as D_PLT_) between these two values (D_PLT_ = PLT_peak_ − PLT_pre_) was calculated. Based on corresponding survival data, we searched for an optimal cut-point of D_PLT_ by x-tile software (Version: 3.6.1, Copyright Yale University 2003–05) [14, 15] to further distinguish prognosis. To avoid statistical bias introduced by ‘multiple cut-off selection’, a cross validation was conducted to test the robustness of the established cut-point [16, 17] (See ‘Result’ part and Fig. 1).

Among the 67 patients, single-course-CR patients (43 cases) were expected to have less marrow function impairment than multiple-course-CR patients (24 cases). We compared the mean of D_PLT_ and PLT_peak_ between the two groups. As we included patients needing 1 to 3 cycles to achieve MRD-negative CR in our study, we first tested whether or not there was significant difference of prognosis between single and multiple-course-CR patients. Secondly, we isolated the 43 single-course-CR patients and determine the optimal cut-point of D_PLT_ by,the same method as above. We compared the predictive strength of D_PLT_ and the number of courses to achieve CR.

After establishing the cut-point, survival analysis was conducted to test whether there is significant difference of PFS or OS between high and low D_PLT_ groups, and whether D_PLT_ is an independent indicator for AML prognosis.

### Survival analysis

PFS is defined as the time from the date of achieving the first MRD-negative CR to the date of relapse or death (with no definite record of relapse). OS is defined as the time from the date of achieving the first MRD-negative CR to the date of death. The optimal cut-point of D_PLT_ was established by x-tile software (Version: 3.6.1, Copyright Yale University 2003–05), then a cross validation analysis as reported by Faraggi and Simon [17] was applied to test its significance. Specifically, a two-folded cross-validation was conducted. The whole dataset was randomly split into two subsets with equal size (Subset A and B). In subset A, we searched for the cutoff value with minimal two-sided log-rank *p*-value. Using this value, we divided subset B into two groups (‘above cut-point’ group and ‘below cut-point’ group). Similarly, in subset B we searched for the cutoff value with minimal two-sided log-rank *p*-value, and divided subset A into ‘above cut-point’ group and ‘below cut-point’ group according to this value. Now every patient in the whole dataset was assigned into either ‘above cut-point’ group or ‘below cut-point’ group. Then the log-rank Chi-square value and corresponding *p*-value stratified by subset A and B was calculated to test the significance of the established cut-point.

Based on the cut-point, the 67 patients were divided into 2 subgroups (high-D_PLT_ and low-D_PLT_). Categorical baseline features of the two subgroups were compared using χ^2^ test/Pearson chi-square test, whereas numerical ones were compared using a non-parametric Mann-Whitney U-test. Survival data was analyzed with Kaplan–Meier methodology, and the differences between groups were evaluated with a log-rank test. Cox multivariate regression analysis was used to test whether or not D_PLT_ is an independent predictor of PFS or OS after considering some potential prognostic covariates, including gender, age, cytogenetic risk, the number of chemotherapy courses needed to achieve MRD-negative CR, and consolidation regimen.

## Results

### Patient characteristics

Clinical and laboratory information of the 67 included patients at diagnosis is shown in Table 1. Among the 67 patients, 33 were male, and 34 were female. Median age at diagnosis was 40.0 years (range of 3–69 years). According to 2016 WHO diagnosis and risk stratification system of AML [2], 12 patients were classified as favorable cytogenetics, 13 as adverse cytogenetics, and the remaining 42 as intermediate risk. At the time of diagnosis, median WBC count was 21.2 X 10^9^/L (range of 0.7–296.4), median hemoglobin level was 77 g/L (range of 39–144), and median platelet level was 43 X 10^9^/L (range of 3–282) (Table 1). Forty-three patients achieved MRD-negative CR after 1 course of induction therapy, twenty-one after 2 courses, and three after 3 courses. During marrow recovery after the chemotherapy course that induced the first MRD-negative CR, median platelet peak value (PLT_peak_) was 389 X 10^9^/L (range of 122–984), and median D_PLT_ was 328 X 10^9^/L (range of 86–981) (Table 1). Mean follow-up time was 29 months (range of 5–90 months). Two-year PFS and OS rates of all the 67 patients were 48.1 and 63.5%, respectively. Three-year PFS and OS rates were 40.6 and 57.4%, respectively.

**Table 1:** Clinical characteristics and laboratory test data of the 67 patients achieving MRD-negative CR

Characteristics	
Age (year)	
Median	40.0
Range	3–69
Sex	
Male	33
Female	34
Initial White cell count (10^9^/L)	
Median	21.2
Range	0.7–296.4
Initial Hemoglobin (g/L)	
Median	77.0
Range	39–144
Initial Platelet Count (10^9^/L)	
Median	43.0
Range	3–282
Bone Marrow Blasts (%)	
Median	66.5
Range	22.9–98
Risk Group (No.)	
Favorable	12
Intermediate	42
Adverse	13
Courses needed to achieve MRD-negative CR	
1	43
2	21
3	3
Consolidation Regimen	
AML-201	32
AML-87	24
HDAra-C	11
Peak Platelet Count (PLT_peak_,10^9^/L)	
Median	389.
Range	122–984
D_PLT_ (10^9^/L)	
Median	328
Range	86–981

### Prognosis analysis for survival

Notice that there are two available survival datasets for determination of the optimal cut-point: PFS and OS. For PFS, the optimal cut-point is D_PLT_ = 212 × 10^9^/L (statistically significant, Chi-square value is 5.833, and *p*-value is 0.016 given by cross validation), while for OS no cut-point with statistical significance was found (p-value is 0.106) (See Fig. 1). We then choose the cut-point as D_PLT_ = 212 × 10^9^/L for following analysis. Based on this value, all the 67 patients were divided into high and low D_PLT_ groups. The characteristics of patients in these two subgroups are shown in Table 2. No significant difference was observed between the two subgroups in terms of distribution of gender, age, cytogenetic risk, consolidation regimen, and the number of chemotherapy courses needed to achieve MRD-negative CR.

**Table 2:** Clinical characteristics and laboratory test data of patients in high D_PLT_ group and low D_PLT_ group (Cutoff value: D_PLT_ = 212 × 10^9^/L)

Characteristics	High D_**PLT**_ Group (*n* = 52)	Low D_**PLT**_ Group (*n* = 15)	***p*** value
Age (year)			
Median	38.5	42.0	0.144
Range	3–69	20–67	
Sex			
Male	24	9	0.345
Female	28	6	
Initial White cell count (10^9^/L)			
Median	18.3	45.3	0.299
Range	0.7–296.41	1.04–149	
Risk Group (No.)			
Favorable	10	2	0.870
Intermediate	32	10	
Adverse	10	3	
Courses needed to achieve MRD-negative CR			
1	36	7	0.092
2	15	6	
3	1	2	
Consolidation Regimen			
AML-87	18	6	0.783
AML-201	26	6	
HD Ara-C	8	3	

We found that patients in high D_PLT_ group had significantly better PFS (2-year PFS rate 56.3% versus 22.2%, 3-year PFS rate 49.7% versus 11.1%; cross validation *p*-value = 0.016.) and better OS, although without statistical significance (2-year OS rate 68.5% versus 45.9%, 3-year OS rate 60.9% versus 45.9%; cross validation p-value = 0.106) (Fig. 1c and d).

Furthermore, by conducting a multivariate analysis of PFS and OS, we found that high D_PLT_ is an independent predictor of better prognosis. For PFS, HR = 2.894, 95% confidence interval 1.320–6.345, *p* = 0.008. For OS, the variables were HR = 3.077, 95% confidence interval: 1.130–8.376, *p* = 0.028 (See Table 3).

**Table 3:** Results of analysis of risk factors for PFS and OS

Factors	PFS				OS			
	univariate		multivariate		Univariate		multivariate	
	HR(95%CI)	*P* value	HR(95%CI)	*P* value	HR(95%CI)	*P* value	HR(95%CI)	*P* value
**Age** **(As a numerical variate)**	1.013 (0.991–1.036)	0.247	1.008 (0.982–1.034)	0.559	1.011 (0.984–1.038)	0.429	0.998 (0.969–1.027)	0.871
**Sex**								
**Male**	1.280 (0.630–2.598)	0.495	1.207 (0.560–2.599)	0.631	1.625 (0.678–3.897)	0.277	1.545 (0.594–4.016)	0.372
**Female**	1.00	–	1.00	–	1.00	–	1.00	–
**Cytogenetics and Molecular Risk**								
**Favorable**	1.00	–	1.00	–	1.00	–	1.00	–
**Intermediate**	1.459 (0.501–4.252)	0.489	1.573 (0.469–5.280)	0.463	4.190 (0.555–31.654)	0.165	2.743 (0.294–25.590)	0.376
**Adverse**	1.538 (0.433–5.462)	0.506	1.809 (0.484–6.768)	0.379	4.011 (0.446–36.050)	0.215	6.744 (0.676–67.256)	0.104
**Number of Induction Therapy Courses to achieve MRD-negative CR**								
**1**	1.00	–	1.00	–	1.00	–	1.00	–
**More than 1**	0.743 (0.359–1.539)	0.424	0.655 (0.286–1.498)	0.316	0.686 (0.284–1.660)	0.404	0.494 (0.188–1.295)	0.151
**Consolidation Regimen**								
**AML-87**	0.798 (0.320–1.987)	0.627	1.019 (0.318–3.266)	0.975	1.274 (0.414–3.921)	0.673	1.858 (0.441–7.820)	0.398
**AML-201**	0.652 (0.252–1.687)	0.377	0.892 (0.320–2.488)	0.827	0.372 (0.093–1.493)	0.163	0.374 (0.088–1.592)	0.183
**HDAra-C**	1.00	–	1.00	–	1.00	–	1.00	–
**D**_**PLT**_								
**Hlgh**	1.00	–	1.00	–	1.00	–	1.00	–
**Low**	3.106 (1.509–6.394)	0.002	2.894 (1.320–6.345)	0.008	2.525 (1.056–6.035)	0.037	3.077 (1.130–8.376)	.0.028

### Comparative analysis of single-course-CR and multiple-course-CR patients

In our study, 43 single-course-CR patients and 24 multiple-course-CR patients were included. No significant difference was found in platelet counts before treatment initiation (*p*-value = 0.663). According to our data, patients achieving MRD-negative CR with 1 course had a significantly higher platelet peak value (PLT_peak_) as well as platelet difference (D_PLT_) compared to patients with more courses. For single-course-CR patients D_PLT_ = (388.0 ± 176.3) × 10^9^/L, while for multiple-course-CR patients D_PLT_ = (300.6 ± 153.5) × 10^9^/L (*p*-value = 0.046). For single-course-CR patients PLT_peak_ = (456.4 ± 161.5) × 10^9^/L, while for multiple-course-CR patients PLT_peak_ = (358.0 ± 165.6) × 10^9^/L (p-value = 0.021). However, no significant difference of PFS or OS was observed between the two groups by Kaplan-Meier method. (*p* = 0.322 for PFS and *p* = 0.309 for OS) .

Next, to clarify if these differences also manifest in differential treatment outcome, we isolated the 43 single-course-CR patients, and searched for an optimal cut-point by x-tile software in this subgroup of patients. Among the 43 single-course-CR patients no statistically significant cut-point was found (*p*-value given by cross validation is 0.157 for PFS and 0.312 for OS). Because significant difference of clinical outcomes was found between high- and low-D_PLT_ subgroups rather than single- and multiple-course-CR subgroups, and there was no evidence for a different distribution of induction course number between high- and low-D_PLT_ subgroups, we speculate that the number of induction cycles to achieve CR is less informative than our new index D_PLT_ concerning AML prognosis.

## Discussion

A whole set of diagnostic methods, including at least morphologic, flow cytometric, cytogenetic, and molecular examination, is necessary to make a precise diagnosis for AML. Pre-treatment information from patients is undoubtedly the footstone to form an overview of the disease and select the proper way of treatment. On the other hand, post-treatment information, such as MRD, can be regarded as “true response” rather than “prediction.” These data helps to adjust the treatment plan according to the real condition of patients [18, 19]. Some related concepts, such as clonal evolution [20], have provided the most profound understanding of the disease. Thus, post-treatment information and instant changes during treatment should be carefully considered. This study focused on post-treatment information.

Some previous studies reported that high blood cell counts after CR (morphological definition) are predictive of longer PFS and OS [21, 22]. In the current study, we strictly selected patients who achieved MRD-negative CR, and examined whether or not a high level of platelet recovery is still predictive of a better prognosis under such a stricter criterion of CR. We speculate that CR is possibly a continuous process with different degrees rather than an “either–or” question. Degree of CR is a reasonable powerful predictor of PFS and OS, which importance should be noted. However, accurate measurement of it is still inaccessible due to limitations of existing methodologies.

In our study, both single-course-CR and multiple-course-CR patients were included, among whom single-course-CR patients had significantly higher PLT_peak_ and D_PLT_ levels. However, survival analysis by Kaplan-Meier method showed no significant difference of PFS or OS between these two subgroups. According to our data, the number of courses to achieve CR is a minor factor of influence, which by itself is not sufficient to distinguish differential clinical outcomes. In contrast, significant difference emerges when we choose D_PLT_ as the prognosis indicator. D_PLT_ appears to be able to stratify for differences in treatment outcome and therefore have prognostic value, whereas the number of induction cycles is less informative in comparison.

To select patients with MRD-negative CR, we had to apply stringent selection criteria, which limited the number of patients included in this study. As a result, some constraints of this study merit attention. Firstly, the size of each group is relatively small, thereby restricting the choice of study designs. An independent validation cohort is absent to test the robustness of the established cut-point. Secondly, the study is retrospectively designed. Our findings require further evaluation and confirmation in a larger-scale prospective study with independent testing and validation cohorts. However, our study provides a clear basis for further investigations of post-treatment response biomarkers.

The underlying mechanism of this phenomenon needs further exploration. Researchers found that marrow failure in AML patients is probably due to a differentiation block impeding hematopoietic stem cells in the leukemic marrow to produce sufficient progenitors, rather than the depletion of hematopoietic stem cells [23, 24]. Many existing studies have also reported the effect of leukemic microenvironment to promote leukemic cell proliferation and survival, and to suppress normal hematopoiesis [25–27]. In addition, AML patients with CRp (complete remission with platelet count < 100 × 10^9^/L) or CRi (complete remission with absolute neutrophil count < 1.0 × 10^9^/L) are observed to have higher MRD levels and poorer outcomes [3]. Based on these findings, we speculate that the degree of platelet count recovery (D_PLT_) may reflect the degree of remission and the improvement of the bone marrow microenvironment for normal hematopoiesis. This view is further supported by our observation that the signal for PFS is stronger compared to OS, suggesting better treatment response, but no ultimate cure (OS is influenced by more factors besides remission degree).

In previous studies, the platelet peak value after CR, similar to PLT_peak_ in our study, is the main study index. Here we chose D_PLT_ as the prognostic indicator of clinical outcomes. We also explored the relationship between PLT_peak_ and PFS/OS for comparison. Again patients in high PLT_peak_ group were found to have a better PFS and a better OS, but without statistical significance by cross validation. Our results showed that D_PLT_ rather than PLT_peak_ stratify differences of clinical outcomes better, especially for PFS. D_PLT_ seems a better prognosis indicator than PLT_peak_ alone. Theoretically, by calculating the difference of platelet count between “before treatment” and “after CR” of the same patient, D_PLT_ may at least partly reduce the bias caused by individual difference in overall platelet counts.

In this study, we determined the cut-point as D_PLT_ = 212 × 10^9^/L and test its significance by a cross validation analysis. We want to emphasize that the optimal cut-point cannot be easily determined. Peripheral blood counts are influenced by many factors; hence, the normal platelet level of the patients before suffering from AML (though difficult to get) may also be valuable. Whether or not a positive correlation exists between platelet recovery degree and remission degree still remains unclear. Further exploration and careful clinical observation are needed to discover the true biological meaning of this phenomenon.

## Conclusions

This study demonstrated that the degree of platelet recovery after achieving the first MRD-negative CR (D_PLT_) is a potential predictor of clinical outcomes, especially for PFS, in CR patients. Higher D_PLT_ value is associated with longer PFS and OS. Our findings may help to develop simple methods for AML prognosis evaluation, although larger-scale prospective studies with independent testing and validation cohorts are required to further test our results.

## Data Availability

The datasets during and/or analysed during the current study available from the corresponding author on reasonable request.

## Abbreviations

AML: Acute myeloid leukemia
MRD: Minimal residual disease
CR: Complete remission
PFS: Progression free survival
OS: Overall survival
PLT_pre_: Peripheral platelet counts before initial treatment
PLT_peak_: Peripheral platelet peak value after marrow recovery following the chemotherapy course inducing the first MRD-negative CR
D_PLT_: Difference between PLT_peak_ and PLT_pre_
MFC: Multiparametric flow cytometry
CRi: Complete remission with absolute neutrophil count < 1.0 × 10^9^/L
CRp: Complete remission with platelet count < 100 × 10^9^/L
